# Boosted electrochemical performance of magnetic caterpillar-like Mg_0.5_Ni_0.5_Fe_2_O_4_ nanospinels as a novel pseudocapacitive electrode material

**DOI:** 10.1038/s41598-023-35014-w

**Published:** 2023-05-15

**Authors:** Matin Shirzad Choubari, Soghra Rahmani, Jamal Mazloom

**Affiliations:** Department of Physics, Faculty of Science, University of Guilan, Namjoo Avenue, P.O. Box 4193833697, Rasht, Iran

**Keywords:** Electrochemistry, Energy, Materials science, Materials for energy and catalysis, Magnetic properties and materials

## Abstract

Ni-incorporated MgFe_2_O_4_ (Mg_0.5_Ni_0.5_Fe_2_O_4_) porous nanofibers were synthesized using the sol–gel electrospinning method. The optical bandgap, magnetic parameters, and electrochemical capacitive behaviors of the prepared sample were compared with pristine electrospun MgFe_2_O_4_ and NiFe_2_O_4_ based on structural and morphological properties. XRD analysis affirmed the cubic spinel structure of samples and their crystallite size is evaluated to be less than 25 nm using the Williamson–Hall equation. FESEM images demonstrated interesting nanobelts, nanotubes, and caterpillar-like fibers for electrospun MgFe_2_O_4_, NiFe_2_O_4_, and Mg_0.5_Ni_0.5_Fe_2_O_4_, respectively. Diffuse reflectance spectroscopy revealed that Mg_0.5_Ni_0.5_Fe_2_O_4_ porous nanofibers possess the band gap (1.85 eV) between the calculated value for MgFe_2_O_4_ nanobelts and NiFe_2_O_4_ nanotubes due to alloying effects. The VSM analysis revealed that the saturation magnetization and coercivity of MgFe_2_O_4_ nanobelts were enhanced by Ni^2+^ incorporation. The electrochemical properties of samples coated on nickel foam (NF) were tested by CV, GCD, and EIS analysis in a 3 M KOH electrolyte. The Mg_0.5_Ni_0.5_Fe_2_O_4_@Ni electrode disclosed the highest specific capacitance of 647 F g^−1^ at 1 A g^−1^ owing to the synergistic effects of multiple valence states, exceptional porous morphology, and lowest charge transfer resistance. The Mg_0.5_Ni_0.5_Fe_2_O_4_ porous fibers showed superior capacitance retention of 91% after 3000 cycles at 10 A g^−1^ and notable Coulombic efficiency of 97%. Moreover, the Mg_0.5_Ni_0.5_Fe_2_O_4_//Activated carbon asymmetric supercapacitor divulged a good energy density of 83 W h Kg^−1^ at a power density of 700 W Kg^−1^.

## Introduction

The ever-increasing worldwide demand for energy is urging the development of energy storage devices and materials with impressive specific capacitance and brilliant cycle stability, such as supercapacitors^1^. One-dimensional (1D) nanofibers providing numerous active sites for ion absorption are potentially one of the best options as an energy storage electrode owing to their magnificent morphologies^1,2^. Electrospinning is an efficient, cost-effective, controllable in diameter, convenient, and rapid method to fabricate different kinds of one-dimensional nanostructures such as nanofibers, nanobelts, and nanotubes with distinguished performances like excellent cycling stability, notable capacity, and suitable ionic conductivity. Various criteria, such as solution parameters (precursors, viscosity, and solvent), flow rate, applied voltage, heating rate, and temperature, have a significant influence on the morphology of electrospun nanostructures^3^. Metal spinel ferrites, generally known as MFe_2_O_4_ (M: a divalent metal ion), which M and Fe ions may locate at both tetrahedral and octahedral positions in a cubic close packing of oxygen, attracted widespread attention in the past few years due to their simplicity of synthesis, high electrical conductivity, low electrical losses and inherent toxicity, physical and chemical stabilities, spontaneous magnetic and electrochemical nature resulting applications in different fields of technology^4,5^. Previously, the supercapacitive behavior of C/CuFe_2_O_4_^6^, Fe_2_O_3_@SnO_2_^7^, and ZnOFe_2_O_4_^8^ nanofibers have been studied extensively.

Magnesium ferrite (MgFe_2_O_4_), possessing a band gap of 2.18 eV, is a well-known *n*-type semiconducting material mainly used as a microwave absorber and lithium-ion batteries due to moderate saturation magnetization and high chemical stability^9–11^. Nickel ferrite (NiFe_2_O_4_) is an *n*-type semiconductor, having low coercivity and high electrical resistivity^12^. Both MgFe_2_O_4_ and NiFe_2_O_4_ possess cubic inverse spinel structures. In inverse spinel structure, a divalent cation (Mg^2+^, Ni^2+^) occupies half of the octahedral B-sites coordination and a trivalent cation (Fe^3+^) locates at tetrahedral A-sites as well as half of the octahedral B-sites^4,13–17^.

NiFe_2_O_4_ and MgFe_2_O_4_ are composed of inexpensive, environmentally safe, and easily accessible materials. Studies have indicated their applicability as electrode material in supercapacitors [18,19,20, and 21]. Their electrochemical behavior is attributed to the presence of electrochemically active multivalent cations^21^. Despite all the interesting properties, the practical applications of these spinels for supercapacitors are still relatively restricted due to unacceptable capacitance and internal resistances. A promising way to improve the electrochemical performance of spinel ferrites is the design of novel ferrite-based nanocomposites. Hybridization by carbon nanomaterial such as graphene^22^ reduced graphene^23,24^, and carbon nanotubes^25^ with high conductivity can boost the specific capacitance of spinel Ferrite. Mixed ferrites are ferrite spinels that are composed of a mixture of two divalent metal ions with varying ratios. The surface properties are considerably affected by the cation distribution of mixed ferrite, making them electrochemically active^26^. Consequently, the ternary ferrites possess the additional composition-dependent synergistic effect of different elements. In preparation of the ternary ferrite-spinel materials, the chemical valence, structural parameters, and radius of the constituent ions should be greatly considered for minimum formation energy and composition tuning in the continuum. Interestingly, both NiFe_2_O_4_ and MgFe_2_O_4_ are isostructural and consist of isovalent Ni^2+^ and Mg^2+^, thus it is exciting to investigate the applicability of ternary Ni-Mg ferrites in the challenging field of energy storage as potential electrode materials with high electrochemical performance. Wongpratat et al. has reported the hydrothermal synthesis of Ni_1-x_Mg_x_Fe_2_O_4_ (x = 0, 0.25, 0.5, 0.75, and 1.00) nanoparticles using aloe vera extract solution. The results revealed that Ni_0.25_Mg_0.75_Fe_2_O_4_ electrodes show a high specific capacitance with cycling stability of 88.79% after a 1000 cycle^21^. Also, enhancing the surface area of the active materials by the fabrication of diverse morphology using various techniques is the most effective strategy to increase the performance of supercapacitors^21^. The physical properties of diverse morphology of MgFe_2_O_4_ and NiFe_2_O_4_ prepared by various methods such as hollow cubes^27^, core–shell^28^, rose-like nanoflower^18^, mesh-like structure^19^, thin film^29^, beehive-like nanosheets^20^, and nanoparticle^21^ have been reported earlier. One-dimensional nanomaterials, prepared via facile and low-cost electrospinning techniques, revealed superior electrochemical performance in supercapacitors owing to their unique morphology and fascinating properties, representing the great potential for boosting the performance of supercapacitors^30^. However, to the best of our knowledge, no reports are available focusing on the supercapacitive behavior of one-dimensional (1D) MgFe_2_O_4_ nanobelts, NiFe_2_O_4_ nanotubes, and ternary spinel Mg_0.5_Ni_0.5_Fe_2_O_4_ nanofibers. In this research, we have investigated the magnetic, optical, and electrochemical properties of these materials fabricated by the sol–gel electrospinning method. The results suggested that Mg_0.5_Ni_0.5_Fe_2_O_4_ caterpillar-like nanofibers can be used as a promising electrode for supercapacitor applications.

## Materials and methods

### Materials

Magnesium nitrate (Mg(NO_3_)_2_.6H_2_O, > 99%, Merck), nickel nitrate (Ni(NO_3_)_2_.6H_2_O, > 99%, Merck), iron nitrate (Fe(NO_3_)_3_.9H_2_O, > 99%, Merck), N, N-Dimethylformamide (DMF), Polyvinylpyrrolidone (PVP; MW = 1,300,000, Sigma-Aldrich) and ethanol (99.8%, Merck), carbon black (Alfa Aesar), polyvinylidene difluoride (PVDF, Kynar HSV900, Arkema), N-methyl-2-pyrrolidone (NMP, > 99%, Merck), and potassium hydroxide (KOH, > 85%, chem-lab) were analytical grade and used without further purification. Deionized (DI) water (> 18.4 MΩ cm^−1^) from a Merck Millipore water purification system was used throughout the experiment. A flexible interconnected porous nickel foam with 1.5 mm thickness and a purity > 99% was supplied by Latech Scientific Supply Pte. Ltd. (Singapore).

### Preparation of samples

**Figure 1:**
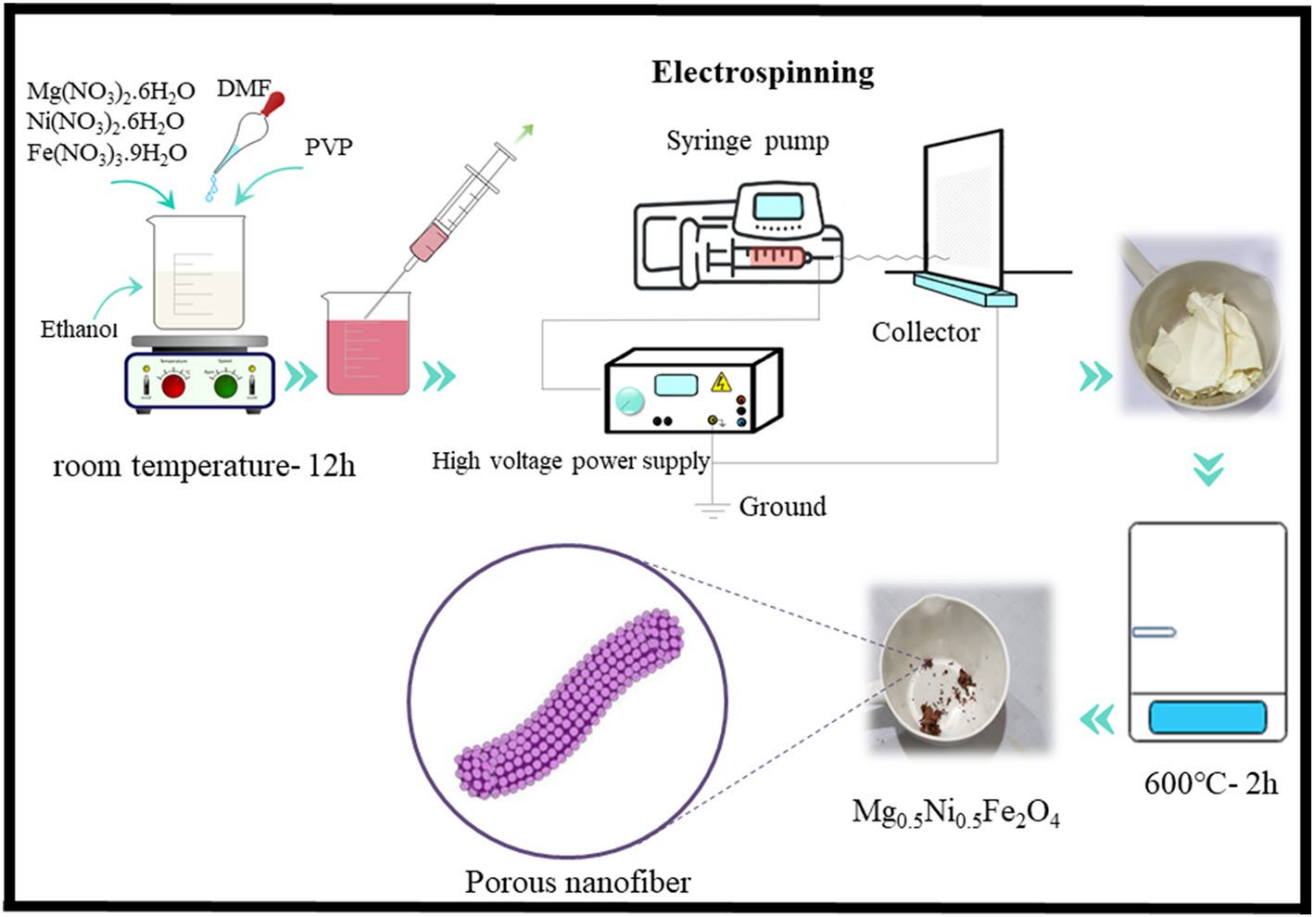
Schematic representation of Mg_0.5_Ni_0.5_Fe_2_O_4_ nanofibers synthesized via sol–gel electrospinning method.

MgFe_2_O_4_ (MFO), NiFe_2_O_4_ (NFO), and Mg_0.5_Ni_0.5_Fe_2_O_4_ (MNFO) nanofibers were fabricated using the sol–gel electrospinning technique. Magnesium ferrite was synthesized by adding 0.2564 g of Mg(NO_3_)_2_.6H_2_O and 0.4039 g Fe(NO_3_)_3_.9H_2_O to 10 mL ethanol/DMF (50:50 w/w) mixed solvents. After that, 1 g of PVP was added dropwise into the above solution and stirred for 12 h. The formed homogeneous sol was loaded into a syringe equipped with a 25 G blunt stainless needle and placed in a syringe pump at a distance of 12 cm from flat aluminum foil as a collector. The syringe pump and collector were connected to a high-voltage power supply, applying a voltage of 17.5 kV. The electrospinning process was done at the rate of 0.5 mL h^−1^ under the controlled condition at room temperature with a relative humidity of 45%. The previous method was repeated to prepare Mg_0.5_Ni_0.5_Fe_2_O_4_ ferrite, where Mg(NO_3_)_2_.6H_2_O (1.282 g), Ni(NO_3_)_2_.6H_2_O (0.1454 g), and Fe(NO_3_)_3_.9H_2_O (0.4039 g) were used as precursors. All of the electrospun samples were dried at 100 °C for 12 h and then calcined at 600 °C in the air for 2 h with a heating rate of 2 °C/min. The schematic representation of Mg_0.5_Ni_0.5_Fe_2_O_4_ nanofibers preparation is shown in Fig. 1.

### Electrode preparation

The synthesized samples as active material (80 wt.%), carbon black (10 wt.%), and PVDF (10 wt.%), were mixed and a slurry was prepared by the addition of N-methyl-2-pyrrolidone (NMP) as a solvent. Then, the prepared slurry was coated onto the nickel foam (1 × 1 cm^2^). Afterward, the electrodes were dried at 80 °C for 3 h. The loaded mass on Ni foam was about 1 mg.

### Material characterizations

Structural properties of prepared nanomaterials were investigated through X-ray diffraction analysis using the X'Pert Pro Philips device. The chemical bonds of nanofibers were studied by Fourier transform infrared analysis using the Alpha—Bruker device. The MIRA3TESCAN-XMU instrument was used to perform field emission scanning electron microscopy and energy dispersive spectroscopy to investigate nanofibers' morphological characteristics and elemental composition, respectively. N_2_ adsorption/desorption isotherms at 77 K were used to evaluate the textural properties of the samples using a BELSORP-mini II instrument. Barrett-Joyner-Halenda (BJH) and Brunauer–Emmett–Teller (BET) techniques were used to measure the pore size distribution and surface area of the samples, respectively. The optical features of samples were explored using the Scinco-S4100 device. Magnetic measurements were done using MDK (Magnetic Daghigh Kavir Co., Iran) device. A three-electrode system, consisting of a prepared electrode, Ag/AgCl, and platinum wire as working, reference, and counter electrodes in a 3 M KOH electrolyte, was used to test the electrochemical efficiency of the synthesized specimens. Using a ZAHNER-ZENNIUM device, cyclic Voltammetry, galvanostatic charge–discharge, and electrochemical impedance spectroscopy (EIS) measurements were conducted over a frequency range of 0.01–10^5^ Hz at an AC amplitude of 10 mV. The Zview software was used to fit the EIS plot.

## Results and discussion

### Structural characterization

**Figure 2:**
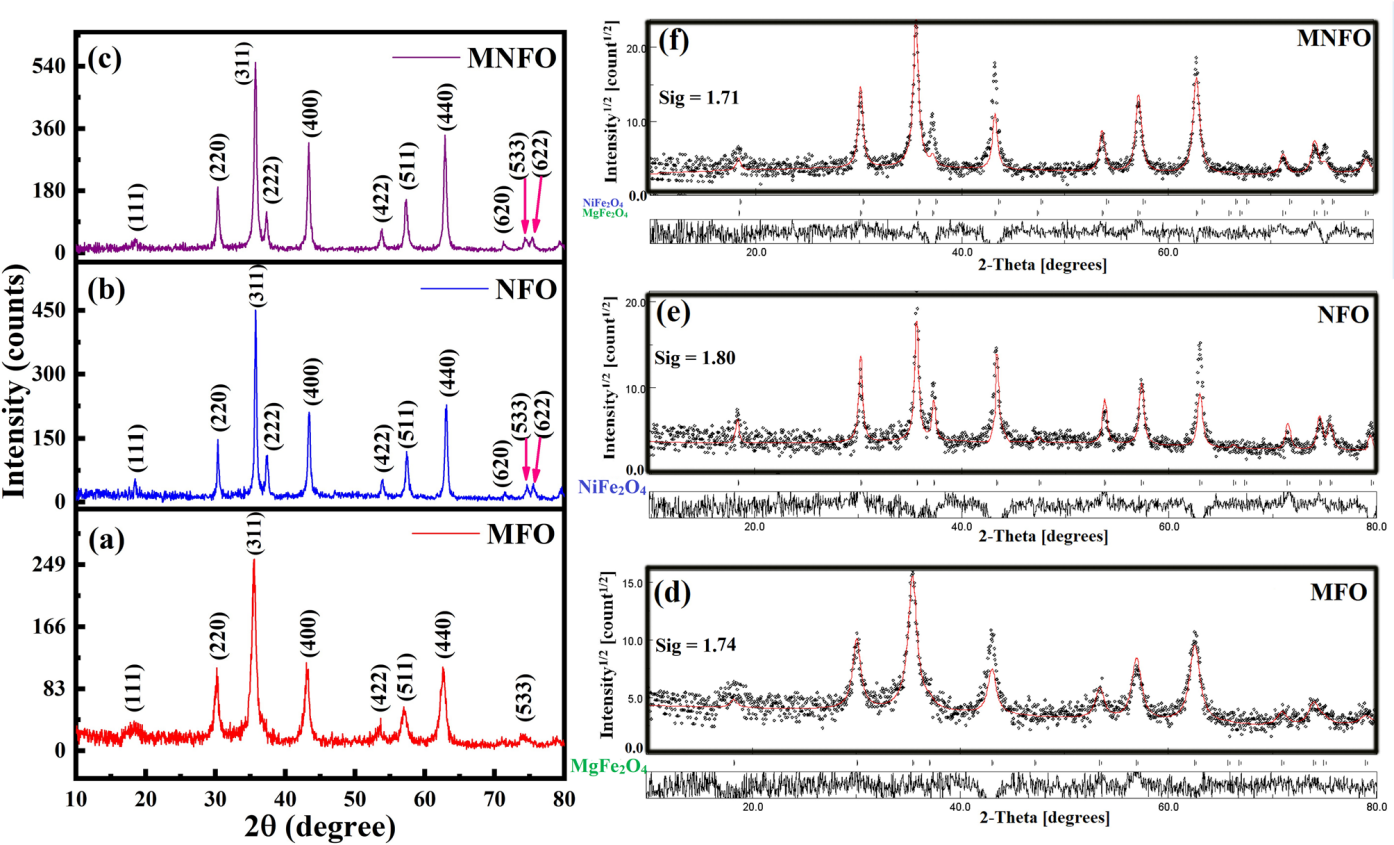
(**a**–**c**) XRD patterns of (**a**) MFO nanobelts, (**b**) NFO nanotubes, and (**c**) MNFO nanofibers, (**d**–**f**) Rietveld fitting of XRD patterns.

The X-ray diffraction patterns of synthesized MgFe_2_O_4_, NiFe_2_O_4_, and Mg_0.5_Ni_0.5_Fe_2_O_4_ nanofibers in the range of 10–80° are demonstrated in Fig. 2a–c. All diffraction peaks are well-indexed as (111), (220), (311), (400), (422), (511), (440), and (533) planes corresponding to single-phase cubic spinel structure of MgFe_2_O_4_ (JCPDS Card No. 36-0398) and NiFe_2_O_4_ (JCPDS Card No. 10-0325)^22,31^. No additional peaks related to any secondary phase were detected in the XRD pattern of synthesized nanofibers, indicating the high-purity phase in samples. On the XRD patterns, Rietveld fitting is conducted using MAUD software to further check the phase purity of the samples. The fitted XRD patterns are shown in Fig. 2d–f. The goodness of fit, Sig, evaluated the fitting quality of the experimental data. The Sig-goodness of those fittings was close to 1 which confirmed that the XRD patterns are compatible with a cubic spinel structure with space group symmetry Fd3m. Furthermore, the Ni^2+^ incorporation in magnesium ferrite not only did not disturb the spinel structure but also the sharp diffraction peaks of the Mg_0.5_Ni_0.5_Fe_2_O_4_ nanofibers suggest the enhanced crystallinity compared to MgFe_2_O_4_ and NiFe_2_O_4_ samples. The lattice constant (*a*) was determined from the interplanar spacing (*d*) for the (311) plane, using the equation for a cubic lattice structure: *a* = *d(h*^2^ + *k*^2^ + *l*^2^*)*^1/2^, where *h*, *k*, and *l* are the Miller indices. The unit cell volume of the samples is denoted in Table 1. As Ni^2+^ was incorporated in magnesium ferrite, the calculated lattice parameter decreased from 8.38 to 8.33 Å due to the substitution of the smaller Ni^2+^ ions (0.69 Å at the octahedral site and 0.55 Å at the tetrahedral site) for the larger Mg^2+^ ions (0.72 Å at the octahedral site and 0.57 Å at the tetrahedral site), which is in line with the results reported for Mg_0.5_Ni_0.5_Fe_2_O_4_ nanoparticles by a hydrothermal route^21^.

**Table 1:** The interplanar spacing (*d*), lattice constant (*a*), cell volume (*V*), the crystallite size (*D*), lattice strain (*ε*), surface area (*S*_BET_), and average pore diameter (*r*_p_).

Sample	Textural parameters						
	*d*_311_ (Å)	*a* (Å)	*V*(Å^3^)	*D* (nm)	***ε***	*S*_BET_ (m^2^ g^−1^)	*r*_p_ (nm)
MFO	2.52	8.38	588.7	6.5	− 0.0025	17.8	3.1
NFO	2.50	8.32	576.5	24.3	0.0006	25.0	5.1
MNFO	2.51	8.33	578.0	22.1	0.0024	40.2	7.3

**Figure 3:**
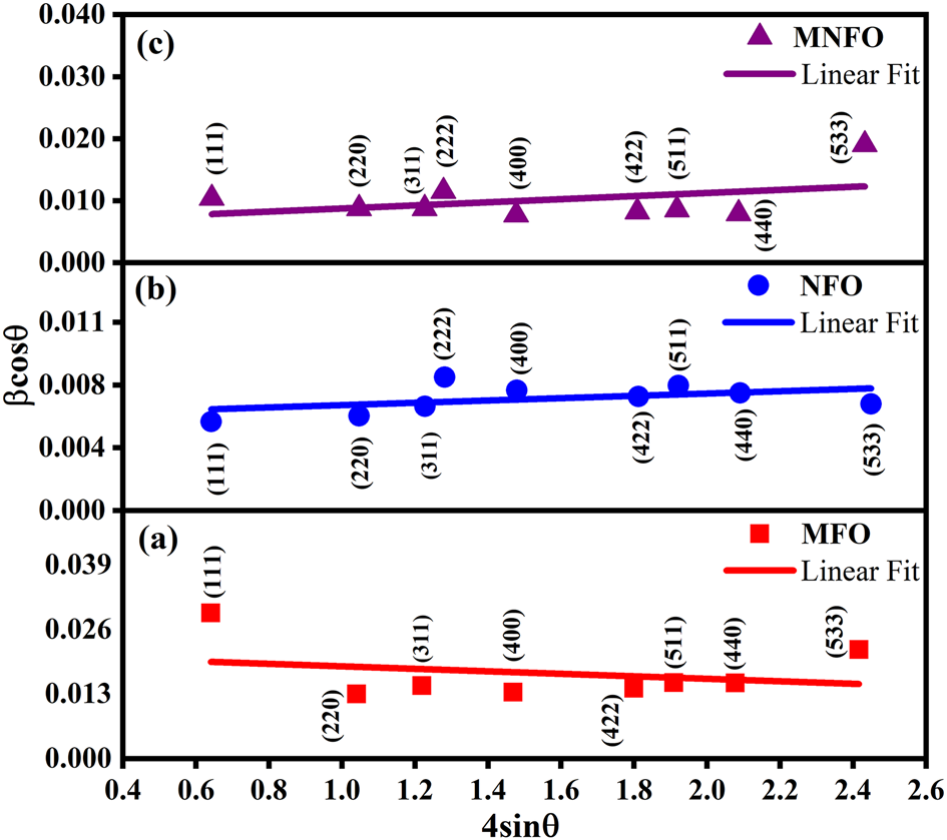
W–H plots of (**a**) MFO nanobelts, (**b**) NFO nanotubes, and (**c**) MNFO nanofibers.

The broadening of the diffraction peak can be assigned to the small crystallite size of the sample, strain, and instrumental factors^32^. The average crystallite size (*D*) and lattice strain (*ε*) of prepared samples are obtained from the intercept and slope of the linear fit of the Williamson–Hall (W–H) plot (Fig. 3), which *β*cos*θ* was plotted with respect to 4sin*θ* according to W–H equation (Eq. 1) for preferred orientation peaks^33^ and the results are listed in Table 1.

1 $$\beta_{hkl} \cos \theta_{hkl} = \frac{K\lambda }{D} + 4\varepsilon \sin \theta_{hkl}$$

where *λ* is the x-ray wavelength, *β* is the width of the peak in radians, *θ* is the Bragg angle, and *K* is a constant which is considered 0.94. The W–H plot revealed a compressive strain for the MFO sample due to lattice shrinkage.

### FTIR study

**Figure 4:**
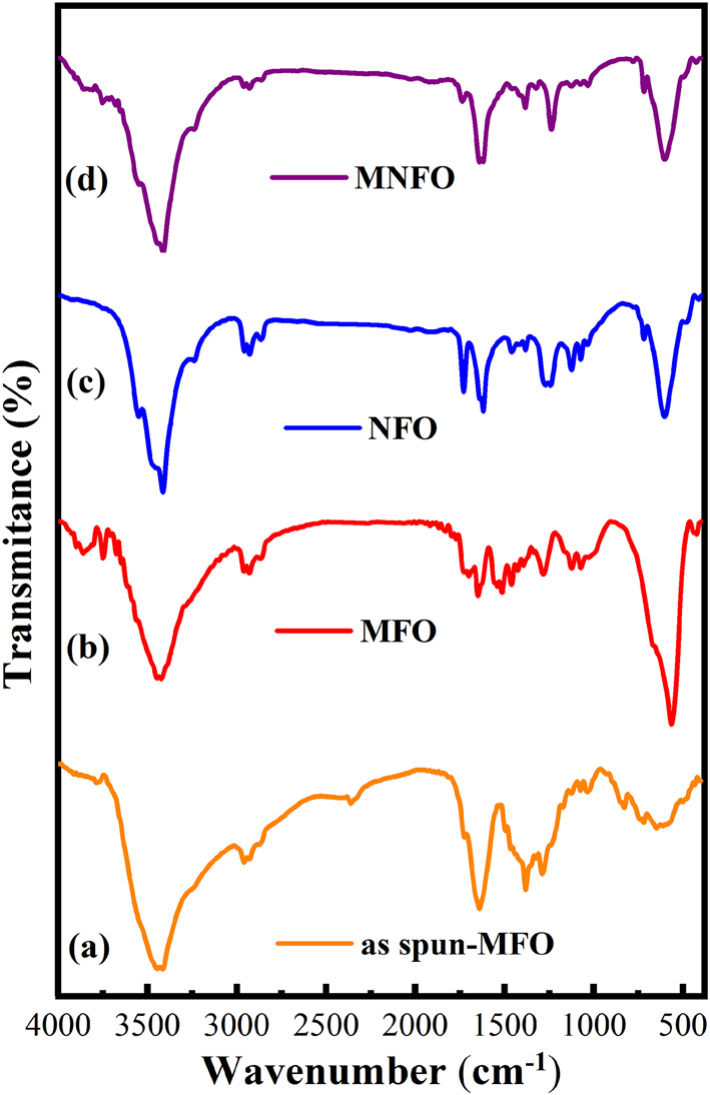
FTIR spectra of (**a**) as-spun MFO, (**b**) MFO nanobelts, (**c**) NFO nanotubes, and (**d**) MNFO nanofibers.

Figure 4 represents the Fourier transform infrared spectra of prepared nanofibers, revealing valuable information about the different functional groups. In general, the spinel ferrite structure consists of two sub-lattices in which divalent ions (Ni^2+^ and Mg^2+^) occupy octahedral B-sites and trivalent ions (Fe^3+^) are equally distributed among tetrahedral A-sites and octahedral B-sites^34^. The bands at 438 and 465 cm^−1^ correspond to the intrinsic stretching vibration of the octahedral sites of Mg-O and Ni–O (ν_o_); also the band at 580 cm^−1^ represents the intrinsic stretching vibration of the tetrahedral site of Fe–O (ν_t_), respectively, confirming the formation of the spinel structure of MgFe_2_O_4_ and NiFe_2_O_4_^22,35^. In the MNFO sample, the metal–oxygen absorption bands slightly shifted with the Ni^2+^ incorporation. This may be due to Mg, Ni, and Fe cations redistribution on both sites. The broad band around 3419 and the less intense band around 1637 cm^−1^ indicated the characteristic vibrational modes of O–H groups and vibrations of the absorbed water molecules, respectively^36^. The bands observed at 2859 and 2937 cm^−1^ disclosed the asymmetric and symmetric stretching vibrations of methylene (–CH_2_) groups, respectively^12^. The band at 1380 cm^−1^ was ascribed to the carboxylate group^37^. The bands in the range of 1000 to 1250 cm^−1^ correspond to nitrate ion traces as well as C–O bonding^22,38^. After calcination, they become weaker. Another band located around 2359 cm^−1^ was reasonably attributed to the anti-symmetrical vibration of carbon dioxide absorbed from the environment, observed only in the as-spun MgFe_2_O_4_ sample^39^.

### Morphological analysis

**Figure 5:**
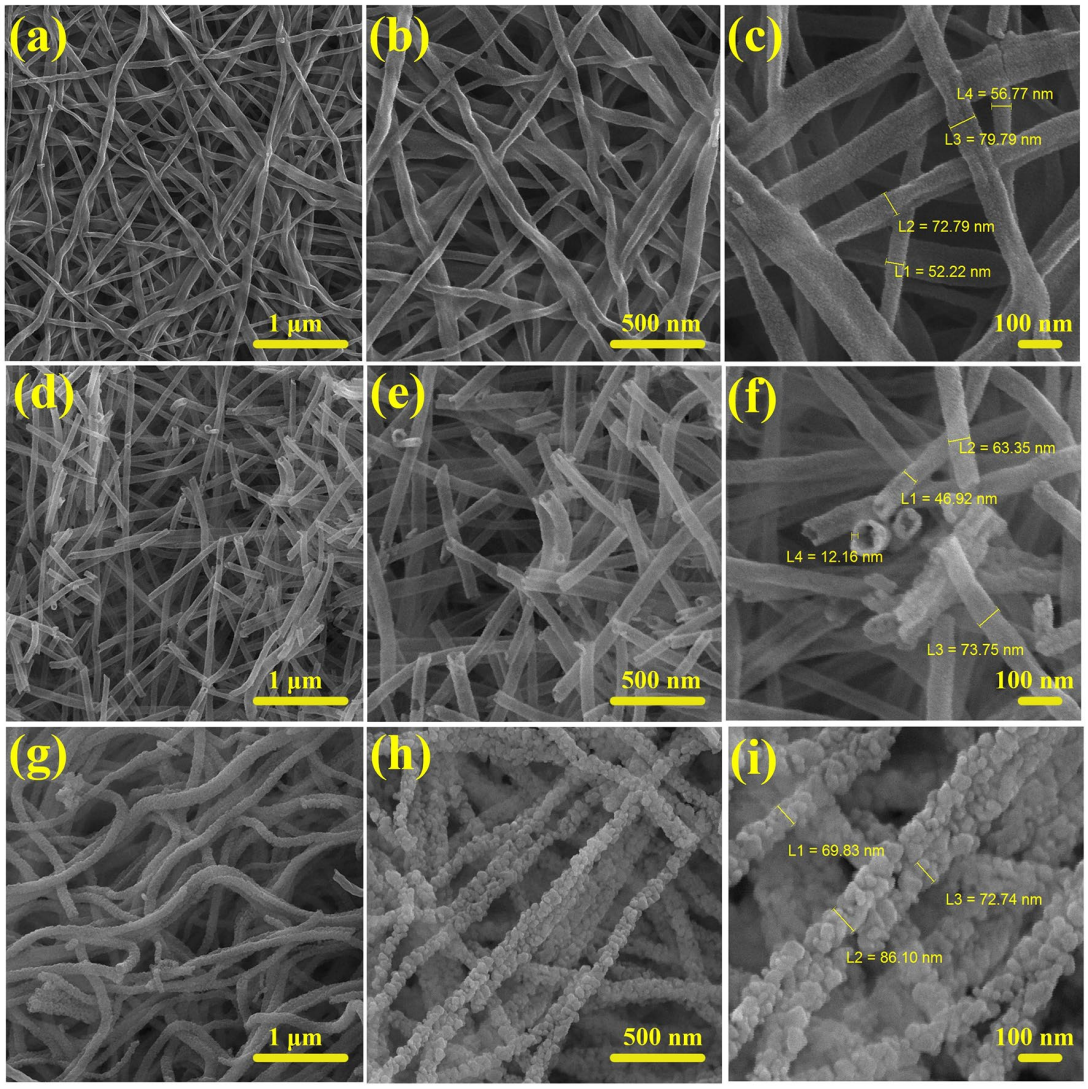
FESEM images of (**a**–**c**) MFO nanobelts, (**d**–**f**) NFO nanotubes, (**g**–**i**), and MNFO nanofibers in low, moderate, and high-magnification.

FESEM images of the prepared MgFe_2_O_4_, NiFe_2_O_4_, and Mg_0.5_Ni_0.5_Fe_2_O_4_ samples calcined at 600 °C are represented at three different magnifications in Fig. 5. It can be found that all of the samples possess one-dimensional arrays. The MgFe_2_O_4_ sample demonstrates long and continuous solid inner belt-like morphology with rectangular cross-sections that possess an average diameter of 65 nm (Fig. 5a–c). In other terms, the collapse of the as-spun polymeric precursor fibers during the calcination led to nanobelts morphology^40^. The NiFe_2_O_4_ depicts short and broken hollow-interior uniform nanotubes (Fig. 5d–f). It has been reported that fast solvent evaporation rate and phase separation during electrospinning tend to the production of hollow nanofibers after calcination^41^. As clearly observed in Fig. 5g–i, the Ni incorporation in MgFe_2_O_4_ causes the formation of roughly porous caterpillar-like nanofibers with numerous grain boundaries. The formation of the rough surface is attributed to the properties of precursor solution, which will be valuable for electrochemical applications where the surface area has a huge impact on determining their performance^41,42^.

**Figure 6:**
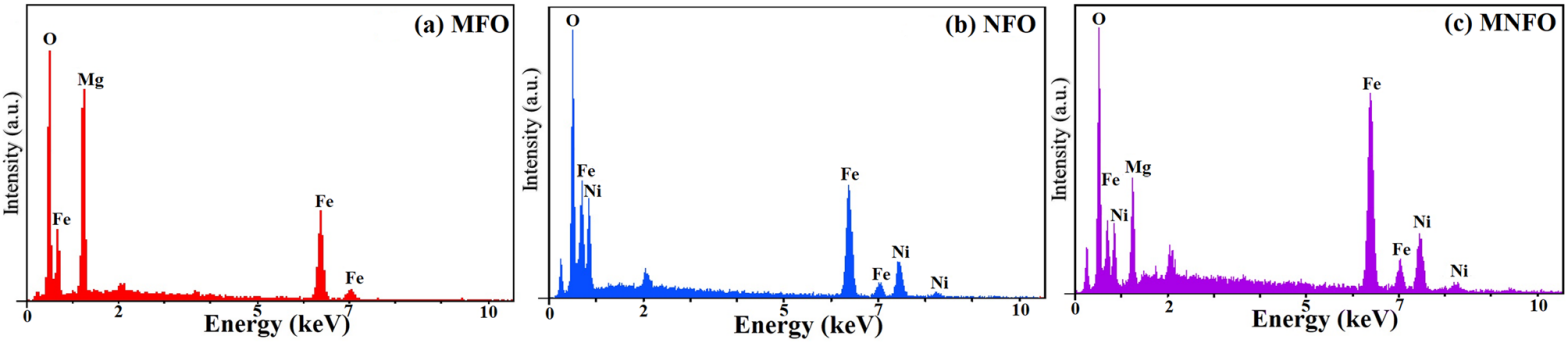
EDS spectra of (**a**) MFO nanobelts, (**b**) NFO nanotubes, and (**c**) MNFO nanofibers.

Figure 6 showed the EDS spectra of prepared samples, which revealed the presence of desired elements such as magnesium (Mg), nickel (Ni), iron (Fe), and oxygen (O) and confirmed the chemical purity of samples^43^. Au peak was normally detected at ~ 2 keV due to the coating of Au thin layer over prepared samples to reduce charging influence^44^.

### Surface area and porosity measurement

**Figure 7:**
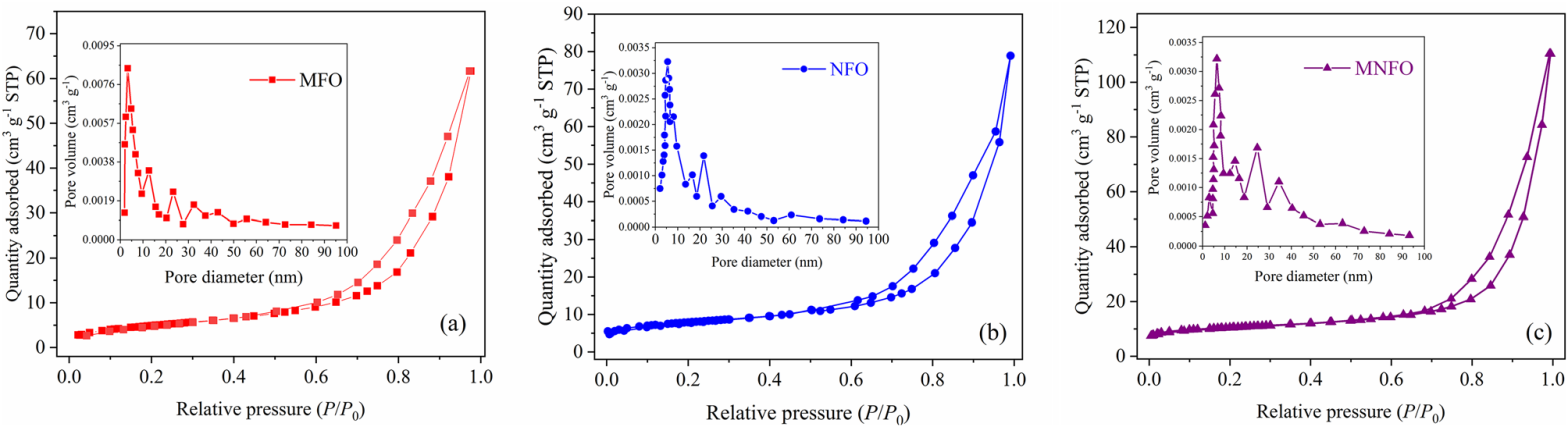
N_2_ adsorption–desorption isotherms of (**a**) MFO, (**b**) NFO and (**c**) MNFO samples (the insets show the corresponding pore size distribution).

The nitrogen adsorption–desorption isotherms of samples are shown in Fig. 7. The isotherm exhibited a typical type-IV behavior with an H3-type hysteresis loop, indicating the presence of mesoporous structure^45^. The formation of mesoporous structure is due to the removal of PVP polymeric matrix after calcination at temperature of 600 °C. The BET specific surface area and average pore diameter of the samples are listed in Table 1. The BET surface area value is calculated as 17.8, 25.0, and 40.2 m^2^ g^−1^ for MFO, NFO, and MNFO samples, respectively. Insets of Fig. 7a–c demonstrates the pore size distribution based on the BJH procedure. The average pore diameter of MNFO fibers was around 7.3 nm. The high surface area of MNFO will be useful for the diffusion of electrolytes, which is vital for the enhancement of electrochemical properties^46^.

### Optical analysis

**Figure 8:**
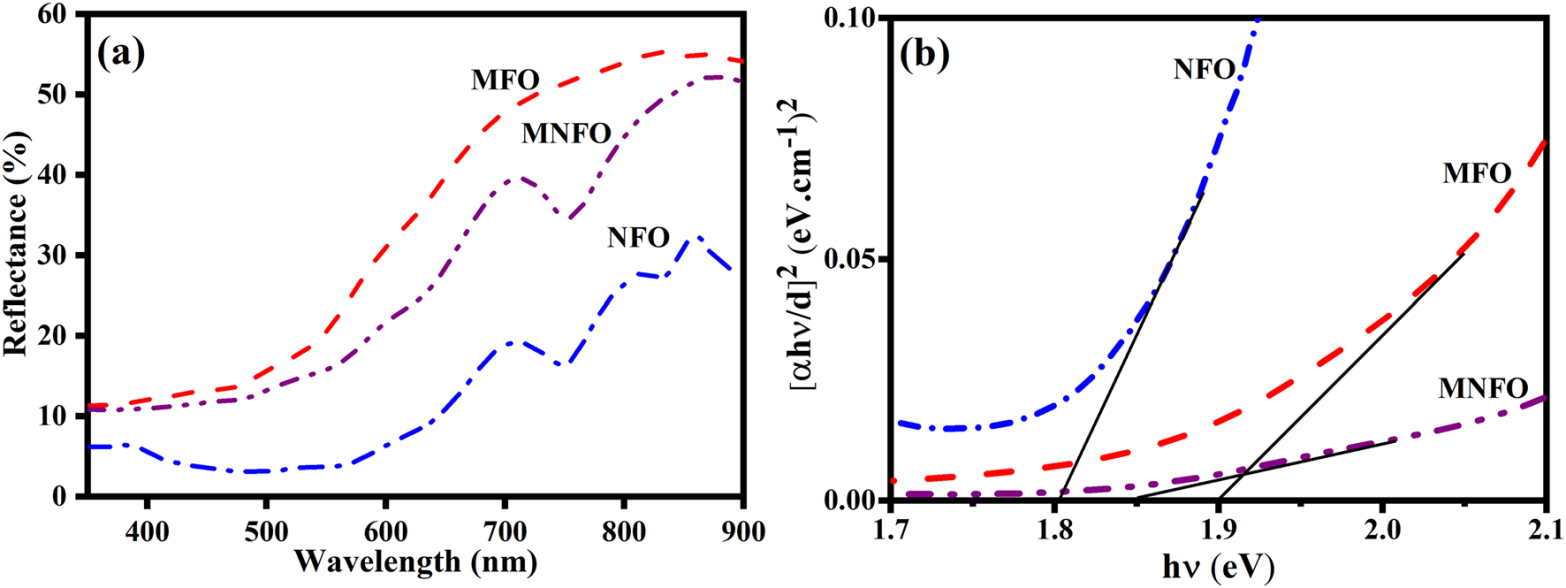
(**a**) DRS spectra, and (**b**) Tauc plots of prepared samples.

In order to investigate the optical properties of prepared nanofibers, the UV–vis-DRS spectra were recorded in the region of 300–900 nm (Fig. 8a). Kubelka–Munk function ($\mathrm{F}\left(\mathrm{R}\right)=\frac{{(1-R)}^{2}}{2R}$) of each sample was utilized to calculate the optical band gap using Tauc equation (Eq. 2)^47^

2 $$\alpha h\nu = A\left( {h\nu - E_{g} } \right)^{n}$$

where *α*, *h*, $\nu$, and* A* represent the material's absorption coefficient proportional to F(R), Planck's constant, the light frequency, and the constant parameters-containing characteristics of the bands, respectively. Also, n = 1/2 is considered to determine the direct optical band gap (E_g_) of MgFe_2_O_4_ and NiFe_2_O_4_, and the plot of (αhν)^2^ versus hν is shown in Fig. 8b. It is known that the structural parameters, crystallite size, and impurities are potential factors that affect the band gap value^44^. The band gap of MFO nanobelts, NFO nanotubes, and MNFO caterpillar-like nanofibers was found to be 1.90, 1.80, and 1.85 eV, respectively. It is seen that the band gap of MNFO nanofibers is narrower than the MFO nanobelts sample because adding nickel in the MNFO preparation process induces inner states in the band gap, providing additional levels between the conduction and the valence bands^17,44^. The calculated band gap value of MNFO nanocomposite lies between the values obtained for the band gap of magnesium ferrite and nickel ferrite due to alloying effect and indicated suitable substitution of Ni ions on Mg sites in MgFe_2_O_4_. The band gap narrowing of magnesium ferrite nanoparticles by Ni substitution was reported by other researchers^11,48^.

### Magnetic analysis

**Figure 9:**
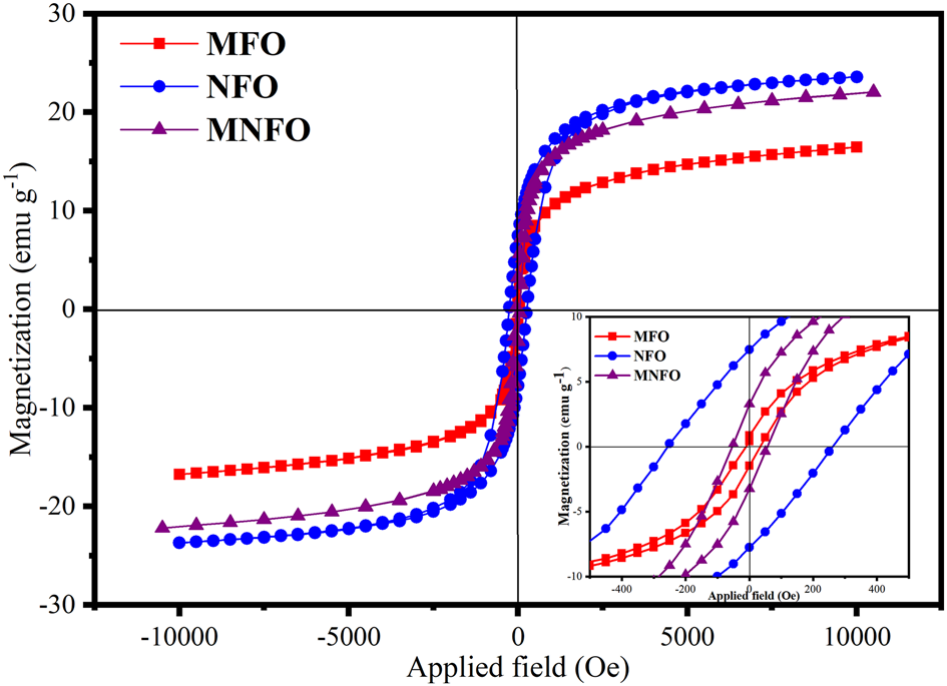
Room temperature VSM plot of MFO nanobelts, NFO nanotubes, and MNFO nanofibers. The inset shows magnified view of M–H curves.

Optimization of the magnetic properties of ferrites has played a decisive role in the application of the magnetic materials group. The vibrating sample magnetometer (VSM) was utilized to measure the magnetization (M) of prepared nanomaterials as a function of the applied magnetic field (H). The hysteresis loops of prepared samples measured at room temperature are demonstrated in Fig. 9. The magnetization was enhanced with an increasing applied magnetic field and saturated at an extremely small value of the applied magnetic field. Also, the small coercivity observed in all hysteresis loops confirms the soft ferromagnetic nature of the synthesized nanofibers^13^. From these hysteresis curves, the value of saturation magnetization (M_s_), remnant magnetization (M_r_), coercivity (H_c_), and the magnetic moment (${\mu }_{B}$) are tabulated in Table 2. The obtained data indicated that the substitution of Mg^2+^ by Ni^2+^ ions in the host MgFe_2_O_4_ lattice had enhanced the value of saturation magnetization from 16.4 to 22.0 emu/g, whereas this value for NiFe_2_O_4_ nanotubes is found to be 23.6 emu/g. The obtained saturation magnetization values of MgFe_2_O_4_ nanobelts and NiFe_2_O_4_ nanotubes are smaller than bulk values of 27 and 47 emu/g, but comparable to the reported values for nanoparticles^49,50^. The reduction of M_s_ relatively to bulk can be attributed to the decreased particle size (enhanced surface/volume ratio) and spin canting at the surface of nanoparticles^49,50^. It is known that the crystallinity, surface imperfection, chemical composition, and cation distribution variation on octahedral and tetrahedral sites have a huge impact on the saturation magnetization of spinel ferrite nanostructures^9,13^. The increase in the M_s_ value of the MFO by Ni incorporation can be attributed to the larger magnetic moments of Ni^2+^ (2.83 ${\mu }_{B}$) as compared to the Mg^2+^ (0 ${\mu }_{B}$) at the octahedral site^51^. The magnetic moment (${\mu }_{B}$) is calculated using the ${\mu }_{B}=\frac{M \times {M}_{s}}{5585}$ equation, where *M*_s_ and *M* represent the saturation magnetization and the molecular weight of the sample^52^ (See Table 2). Moreover, the coercive field, H_c_, was increased from 0.40 for the MgFe_2_O_4_ nanotubes to 3.26 Oe for the MNFO sample. The increase of the surface anisotropy of small crystallites contributed to the enhancement of coercivity^51,53^. This sort of increase in saturation magnetization for nickel-substituted magnesium ferrite has also been reported earlier^54,55^.

**Table 2:** Saturation magnetization (M_s_), remanence (M_r_), coercivity (H_c_), and magnetic moment (${\mu }_{B}$) of (a) MFO nanobelts, (b) NFO nanotubes, and (c) MNFO nanofibers.

Sample	Magnetic parameters			
	M_s_ (emu/g)	M_r_ (emu/g)	H_c_ (Oe)	${\mu }_{B}$
MFO	16.4	0.4	34.3	0.589
NFO	23.6	7.4	261.9	0.992
MNFO	22.0	3.2	56.9	1.019

### Electrochemical measurements

**Figure 10:**
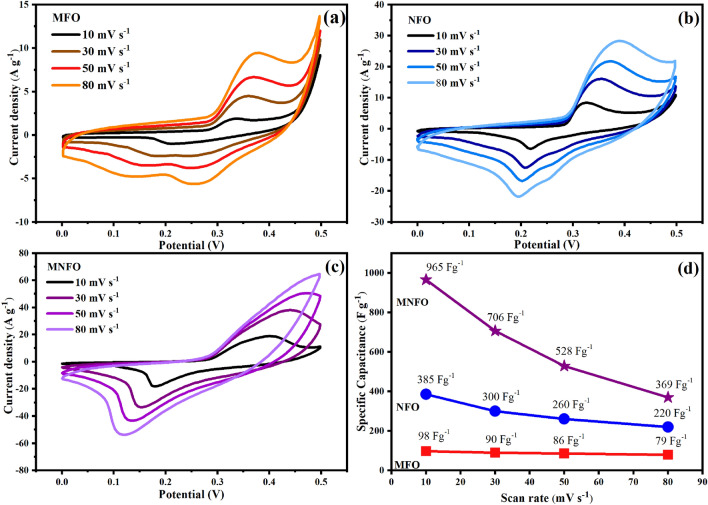
CV plots of (**a**) MFO nanobelts, (**b**) NFO nanotubes, and (**c**) MNFO nanofibers. (**d**) The specific capacitance of samples at scan rates of 10, 30, 50, and 80 mV s^−1^.

The electrochemical performance of MgFe_2_O_4_, NiFe_2_O_4_, and Mg_0.5_Ni_0.5_Fe_2_O_4_ electrodes was tested using a three-electrode system in 3 M KOH. The Cyclic Voltammetry (CV) of prepared samples at various scan rates of 10, 30, 50, and 80 mV s^−1^ with a potential window of 0–0.5 V is shown in Fig. 10a–c. The CV profiles with distinct anodic and cathodic redox peaks demonstrate the supercapacitive nature of prepared nanomaterials. As it is known, the enhancement of ion-electrode interaction (diffusion–reaction at the electrolyte and electrode interface) leads to excellent capacitive behavior ^56^. The energy storage mechanism of prepared samples is suggested by the following reactions^57,58^:

3 $${\text{MFe}}_{2} {\text{O}}_{4} { } + {\text{ H}}_{2} {\text{O }} + {\text{ OH}}^{ - } \leftrightarrow 2{\text{FeOOH}} + {\text{MOOH}} + {\text{ e}}^{ - } { },{ }\left( {{\text{M}} = {\text{Ni}},{\text{ Mg}}} \right)$$

4 $${\text{MOOH}} + {\text{OH}}^{ - } \leftrightarrow {\text{MO}}_{2} + {\text{ H}}_{2} {\text{O}} + {\text{ e}}^{ - } ,$$

5 $${\text{FeOOH}} + {\text{ H}}_{2} {\text{O }} \leftrightarrow {\text{Fe}}\left( {{\text{OH}}} \right)_{3} \leftrightarrow \left( {{\text{FeO}}_{4} } \right)^{2 - } + 3{\text{e}}^{ - }$$

The shape of the cyclic voltammogram of samples remains unchanged as the sweep rate increases from 10 to 80 mV s^−1^_,_ revealing excellent electrochemical reversibility and prominent high-rate performance. However, the shifts of redox peaks towards lower/higher potentials may be attributed to the polarization effect. The specific capacitance from the CV profile was calculated according to the following equation:

6 $$C_{sp} = \frac{\smallint IdV}{{{\text{m}}\upnu \Delta V}}$$

where C_sp_, $\smallint IdV$, m, $\nu$, and ∆V denote the specific capacitance (F g^−1^), the integrated area under the CV plot, the mass of active material (g), the scan rate (V s^−1^), and the potential window (V), respectively^59^. The specific capacitance values of MgFe_2_O_4_, NiFe_2_O_4_, and Mg_0.5_Ni_0.5_Fe_2_O_4_ at the scan rate of 10 mV s^−1^ were 98, 385, and 965 F g^−1^, respectively, as shown in Fig. 10d. The specific capacitance is significantly influenced by the scan rate enhancement. The calculated data of C_sp_ exhibited a higher specific capacitance at lower scan rates. This implies that the electrolyte ions had sufficient time to penetrate and access all the inner microstructures of the electrode material for charge storage^60^.

**Figure 11:**
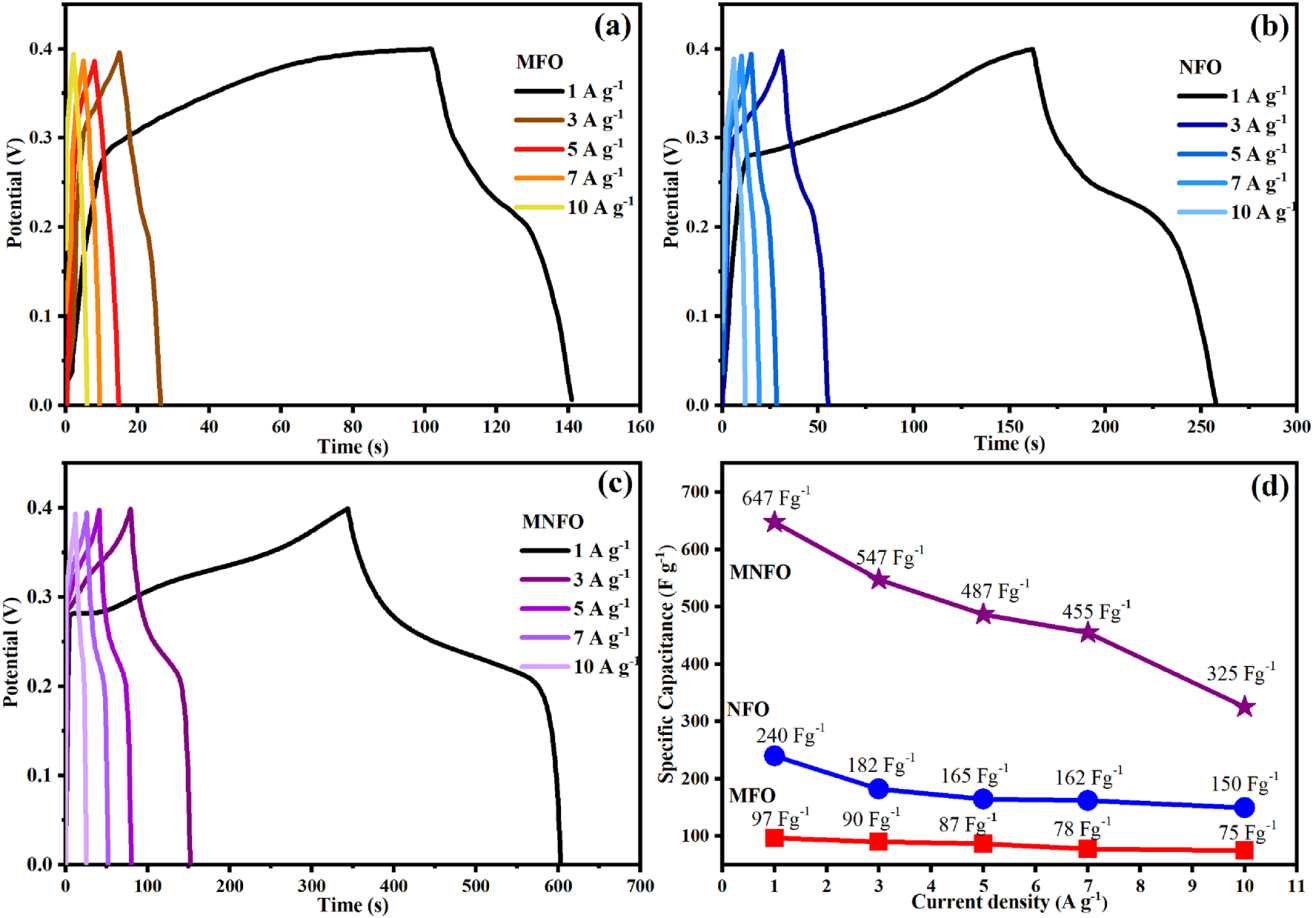
GCD plots of (**a**) MFO nanobelts, (**b**) NFO nanotubes, and (**c**) MNFO nanofibers. (**d**) The specific capacitance at the current density of 1, 3, 5, 7, 10 A g^−1^.

The galvanostatic charge–discharge (GCD) profiles of prepared nanomaterials were recorded at a current density of 1, 3, 5, 7, and 10 A g^−1^ demonstrated in Fig. 11a–c. To prevent an oxygen evolution reaction (OER) at a higher potential during the charging process in an aqueous electrolyte, the GCD test voltage was set in the range of 0–0.4 V. The specific capacitance was calculated from the GCD profile using the following equation:

7 $$C_{sp} = \frac{{{\text{I}} \Delta t}}{{{\text{m}} \Delta V}}$$

where C_sp_, I, ∆t, m, and ∆V denote the current (A), the time of a full discharge (s), the mass of the active material (g), and the potential window (V)^59^. The specific capacitance of prepared samples at various current densities of 1, 3, 5, 7, and 10 A g^−1^ is exhibited in Fig. 11d. The specific capacitance values of MgFe_2_O_4_, NiFe_2_O_4_, and Mg_0.5_Ni_0.5_Fe_2_O_4_ at the current density of 1 A g^−1^ were obtained 97, 240, and 647 F g^−1^, respectively, which declined to 75, 150, and 325 F g^−1^ at the current density of 10 A g^−1^ due to reduced accessibility of active sites in high diffusion rate.

In both CV and GCD tests, the significantly high specific capacitance value of the MNFO samples is attributed to two main convincing reasons: (i) By adding nickel into the magnesium ferrite, the Ni^2+^ ions enhanced the electrochemical activities by enriching the redox states, and the whole process prominently improved the electrochemical chemical performance of the material^61–63^. (ii) The unique morphology of porous nanofibers, including high surface area and grain boundaries, played a major role in the electrochemical activities and capacitive energy storage of prepared materials^64,65^. The electrochemical behavior of nickel or magnesium-based ferrite has also been reported in the literature and some of them are listed in Table 3, indicating that obtained specific capacitance values are higher than those reported earlier by some authors.

**Table 3:** The comparative representation of evaluated specific capacitances (C_sp_) and cyclic Stability of prepared electrodes with other literature results.

Sample	Morphology	Preparation method	Electrolyte	Current density/scan rate	C_sp_ (F g^−1^)	Cyclic Stability (%)	Ref
MgFe_2_O_4_	Rose nanoflowers	Hydrothermal	3 M KOH	1 A g^−1^	250	139.5 (2000 cycles)	^18^
MgFe_2_O_4_	Nanoparticles	Hydrothermal	6 M KOH	1 A g^−1^	153	71.76 (1000 cycles)	^21^
MgFe_2_O_4_	Nanobelts	Electrospinning	3 M KOH	1 A g^−1^	97	81 (3000 cycles)	This work
NiFe_2_O_4_	Nanoparticles	Solvothermal	1 M KOH	1A g^−1^	386	73 (700 cycles)	^19^
NiFe_2_O_4_	Nanosheets	Electrodeposition	1 M KOH	1 A g^−1^	560	95.3 (10,000 cycles)	^20^
NiFe_2_O_4_	Nanospheres	Precipitation	0.5 M KOH	1 A g^−1^	137.2	100 (100 cycles)	^66^
NiFe_2_O_4_	Pompon flower	Vapor deposition	3 M KOH	1 A g^−1^	168.5	71.4 (10,000 cycles)	^67^
NiFe_2_O_4_	Nanoparticles	Solvothermal	3 M KOH	2 mV s^−1^	109.26	< 90 (1000 cycles)	^68^
NiFe_2_O_4_	Nanotubes	Electrospinning	3 M KOH	1 A g^−1^	240	87 (3000 cycles)	This work
Ni_0.6_Mn_0.4_Fe_2_O_4_	Nanoparticles	Hydrothermal	3 M KOH	0.5 A g^−1^	766	95 (1500 cycles)	^17^
Ni_0.5_Mg_0.5_Fe_2_O_4_	Nanoparticles	Hydrothermal	6 M KOH	1 A g^−1^	46.49	96.28 (1000 cycles)	^21^
Co_0.5_Ni_0.5_Fe_2_O_4_	Nanoparticles	Hard template	1 M KOH	5 mV s^−1^	113	120.05 (1000 cycles)	^69^
Zn doped MgFe_2_O_4_	Nanoparticles	Sol–gel citrate	1 M Na_2_SO_4_	1 mA cm^−2^	484.6	–	^70^
MgFe_2_O_4_/ZnMn_2_O_4_	Nanoparticles	Sol–gel	3 M KOH	1 A g^−1^	502	108.9 (1000 cycles)	^71^
NiFe_2_O_4_@CoFe_2_O_4_	Nanofibers	Electrospinning	3 M KOH	1 A g^−1^	480	87 (2000 cycles)	^72^
Mg_0.5_Ni_0.5_Fe_2_O_4_	Nanofibers	Electrospinning	3 M KOH	1 A g^−1^	647	91 (3000 cycles)	This work

**Figure 12:**
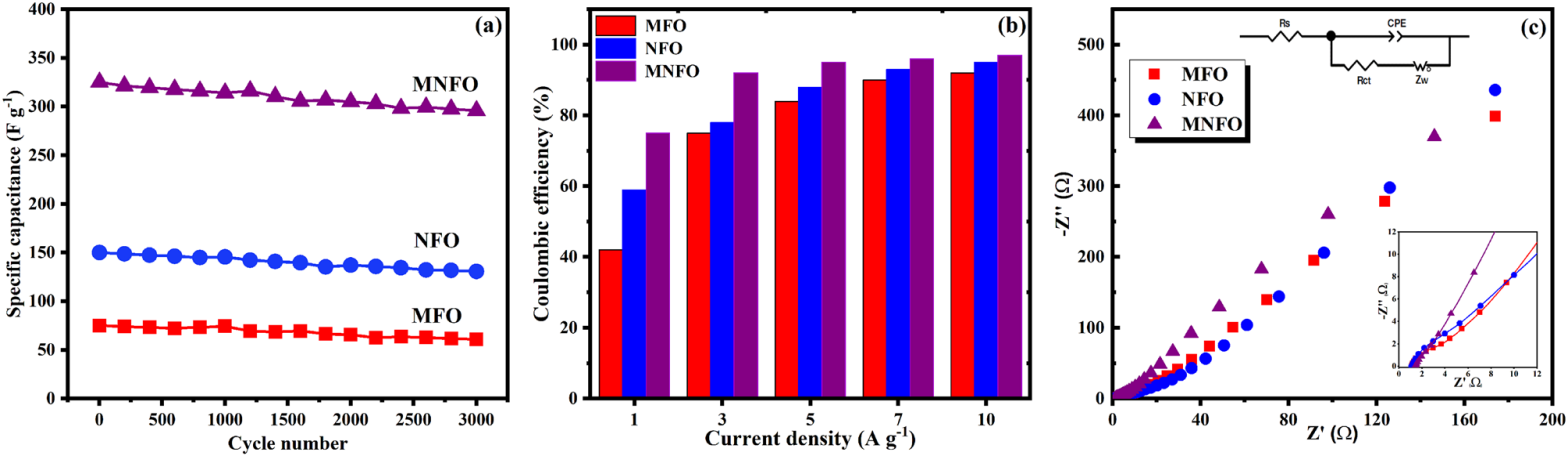
(**a**) Cycling stability of samples at the current density of 10 A g^−1^ after 3000 cycles. (**b**) Coulombic efficiency at the current densities of 1, 3, 5, 7, and 10 A g^−1^. (**c**) EIS plots MFO nanobelts, NFO nanotubes, and MNFO nanofibers, insets show the curves in the high-frequency range and the equivalent circuit.

Long-term cycling stability as a criterion is also studied (see Fig. 12a). The prepared electrodes of MFO, NFO, and MNFO samples exhibited capacitance retention of about 81, 87, and 91% after 3000 cycles at 10 A g^−1^, respectively. The Coulombic efficiency is also estimated according to the below equation:

8 $$\eta = \frac{{t_{D} }}{{t_{C} }} \times 100$$

where η, ${t}_{D}$, and ${t}_{C}$ represent the Coulombic efficiency, charge time (s), and discharge time (s), respectively^73^. The Coulombic efficiency of prepared samples at various current densities of 1, 3, 5, 7, and 10 A g^−1^ is exhibited in Fig. 12b. The MgFe_2_O_4_ nanobelts, NiFe_2_O_4_ nanotubes, and Mg_0.5_Ni_0.5_Fe_2_O_4_ nanofibers demonstrated high Coulombic efficiency of 92, 95, and 97%, respectively, at the current density of 10 A g^−1^. Herein, the superior Coulombic efficiency of MNFO may be attributed to the appropriate formation of 1D surface area and designed architecture with high surface area, which cause the unique reversibility of the charge–discharge process of the prepared sample^46,74^.

The electrochemical impedance spectroscopy (EIS) measurements of prepared nanomaterials were carried out in the frequency range of 0.01 Hz to 100 kHz.

The Nyquist plot and the equivalent circuit diagram consist of charge transfer resistance (R_ct_), solution resistance (sum of electrolyte ionic resistance, electrode-to-current collector contact resistance, and electrode material intrinsic resistance; R_s_), Warburg resistance (Z_w_), and the constant phase element (CPE) are displayed in Fig. 12c. The intercept and semicircle diameter on the real axis in the Nyquist plot denotes the solution resistance and the Faradaic charge transfer resistance, respectively^46,61^. The R_S_ value of electrodes is very low, allowing the electrolyte to access the electrodes surface efficiently^75^. The R_ct_ of MFO, NFO, and MNFO was calculated to be 6.64, 4.79, and 3.16 Ω, respectively. The lowest R_ct_ value of nickel-substituted magnesium ferrite revealed that Ni incorporation in magnesium ferrite structure facilitates the charge transfer efficiency at the interface of electrode and electrolyte, affirming the higher specific capacitance of this electrode^46,75^. Also, steeper slopes of the plot in the low-frequency regions imply that the Mg_0.5_Ni_0.5_Fe_2_O_4_ sample has lower Warburg resistance (greater ionic conductivity) than other samples. Therefore, nickel incorporation in MgFe_2_O_4_ provided better ionic and electronic conductivity in the Mg_0.5_Ni_0.5_Fe_2_O_4_ sample.

**Figure 13:**
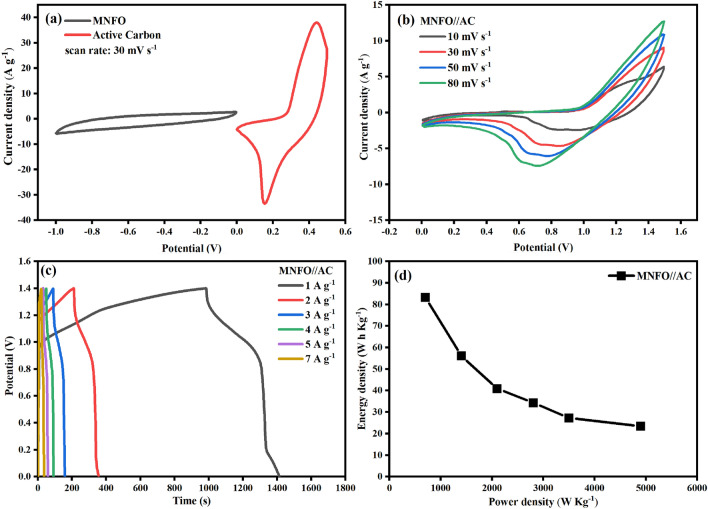
(**a**) CV curves of AC and MNFO//AC at the scan rate of 30 mV s^−1^, (**b**) CV curves of the MNFO//AC cell at various scan rates, (**c**) GCD curves of the MNFO //AC cell at different current densities, and (**d**) Ragone plot of the MNFO//AC cell.

Further exploration is done to study the accurate potential of MNFO sample in real applications, assembling two-electrode cell utilizing MNFO and Active Carbon (AC) as positive and negative electrodes, respectively, in a 3 M KOH electrolyte. At first, the electrochemical properties of AC electrode were investigated by the standard three-electrode system. Figure 13a demonstrates the CV curves of individual AC and MNFO electrodes at a scan rate of 30 mV s^−1^ and complementary potentials within − 1 to 0 V and 0–0.5 V, respectively. As shown in Fig. 13b, the stable potential windows of MNFO//AC asymmetric supercapacitor (ASC) are capable of being extended to 1.5 V, displaying cyclic voltammograms along with weak redox peaks with no polarization. As the scanning rate increases, the enlargement of the CV curves occurs, manifesting the suitable rate performance of the cell. The specific capacitance of MNFO//AC is 306, 206, 150, 126, 100, and 86 F g^−1^ at 1, 2, 3, 4, 5, and 7 A g^−1^ obtained from GCD results (Fig. 13c). The energy density (*E*) and power density (*P*) of MNFO//AC were calculated using Eqs. (9) and (10)^17^, as the Ragone plot is demonstrated in Fig. 13d.

9 $$E = \frac{{C_{sp} \left( {\Delta V} \right)^{2} }}{2 \times 3.6}$$

10 $$P = \frac{3600E}{{\Delta t}}$$

Where, C_sp_ (F g^−1^) is the specific capacitance, ∆V (V) is the working voltage window, and ∆t (s) is the discharge time of the MNFO//AC cell. The results revealed a high energy density of 83 W h Kg^−1^ and a power density of 700 W Kg^−1^ at a current density of 1 A g^−1^.

## Conclusion

In this work, an attempt has been made to achieve the superior electrocapacitive performance from the novel and well-designed ternary Mg_0.5_Ni_0.5_Fe_2_O_4_ spinel ferrite nanofibers compared to pure MgFe_2_O_4_ nanobelts and NiFe_2_O_4_ nanotubes prepared by electrospinning technique. The XRD, FTIR, FESEM, EDS, DRS, and VSM studies are also done to show the maximum functionality of samples. XRD and FTIR results showed the well-crystallized cubic spinel phase and metal–oxygen bonds of the samples on the octahedral and tetrahedral sites, respectively. The optical band gap of Mg_0.5_Ni_0.5_Fe_2_O_4_ was narrower than MgFe_2_O_4_ nanobelts. The enhancement of saturation magnetization and coercivity of MgFe_2_O_4_ nanobelts via Ni^2+^ ions substation was confirmed using the VSM test. The electrochemical study revealed that although the specific capacitance obtained for the pristine magnesium ferrite nanobelts was small, the incorporation of nickel into its structure caused the formation of a novel ternary ferrite with a significant capacitance. The highest specific capacitance of 647 F g^−1^ for Mg_0.5_Ni_0.5_Fe_2_O_4_ with outstanding cycling stability of 91% after 3000 cycles at 10 A g^−1^ was achieved which is far greater than pristine MgFe_2_O_4_ and NiFe_2_O_4_. Furthermore, the Mg_0.5_Ni_0.5_Fe_2_O_4_//Activated carbon asymmetric supercapacitor cell could be cycled reversibly in the high-voltage range of 0 to 1.5 V and divulged intriguing performances with an energy density of 83 W h Kg^−1^ at a power density of 700 W Kg^−1^. Mg_0.5_Ni_0.5_Fe_2_O_4_ electrode with safe and suitable electrochemical performance is promising for practical application in energy storage devices and might play an important role in renewable energy, potentially reducing pollution and decreasing the consumption of hydrocarbon fuels. We hope that this work can open up new possibilities for exploring novel ternary ferrite spinels as electrode materials for application in the energy storage field.

## Data Availability

All data generated or analyzed during this study are included in this published article, and the datasets used and analyzed during the current study are available from the corresponding author upon reasonable request.
